# Involved, inputting or informing: “Shared” decision making in adult mental health care

**DOI:** 10.1111/hex.12601

**Published:** 2017-08-04

**Authors:** Eleanor Bradley, Debra Green

**Affiliations:** ^1^ Institute of Health and Society University of Worcester Worcester UK; ^2^ Research and Innovation Department St George's Hospital Stafford UK

**Keywords:** adherence, family and informal carers, serious mental illness, shared decision making

## Abstract

**Background:**

A diagnosis of serious mental illness can impact on the whole family. Families informally provide significant amounts of care but are disproportionately at risk of carer burden when compared to those supporting people with other long‐term conditions. Shared decision making (SDM) is an ethical model of health communication associated with positive health outcomes; however, there has been little research to evaluate how routinely family is invited to participate in SDM, or what this looks like in practice.

**Objective:**

This UK study aimed to better understand how the family caregivers of those diagnosed with SMI are currently involved in decision making, particularly decisions about treatment options including prescribed medication. Objectives were to

1. Explore the extent to which family members wish to be involved in decisions about prescribed medication
2. Determine how and when professionals engage family in these decisions
3. Identify barriers and facilitators associated with the engagement of family in decisions about treatment.

**Participants:**

Open‐ended questions were sent to professionals and family members to elicit written responses. Qualitative responses were analysed thematically.

**Results:**

Themes included the definition of involvement and “rules of engagement.” Staff members are gatekeepers for family involvement, and the process is not democratic. Family and staff ascribe practical, rather than recovery‐oriented roles to family, with pre‐occupation around notions of adherence.

**Conclusions:**

Staff members need support, training and education to apply SDM. Time to exchange information is vital but practically difficult. Negotiated teams, comprising of staff, service users, family, peers as applicable, with ascribed roles and responsibilities could support SDM.

## 1 INTRODUCTION

There are ~1.5 million people caring for a person with either a mental illness or dementia in the UK, and this number is expected to grow. An estimated 1/3‐2/3s of people with serious mental illness (SMI) live with family, saving the UK economy over £119 billion per year.^1^

Despite their prominent role, the family members of those with SMI remain a socially excluded group.^2^ Families feel marginalized that their expertise is overlooked and are not routinely offered support themselves. Family members who are struggling to provide care without adequate support run the risk of neglecting their own social networks, leaving themselves isolated.^3^ The family members of those diagnosed with SMI appear at greater risk of lower health‐related quality of life and stress‐related illness than either the general population or those caring for people with somatic illness.^4^

Carers of people with SMI utilize a wide range of coping styles including active behavioural strategies, active cognitive style strategies and avoidant style strategies.^5^ Of these strategies, avoidance is the most likely to be associated with burden or distress. In the absence of clear and timely information, family members may employ avoidant rather than active strategies.

Hope is fundamental throughout recovery for those diagnosed with a SMI. Carers have been described as “hope carriers”^6^—as those who remain hopeful even when those they are caring for feel “hopeless.” Understanding family resilience in the face of difficult health experiences is vital.

Living with a long‐term condition presents challenges, but there are particular challenges for those involved in supporting people with a diagnosis of SMI. Clinical heterogeneity is evident, and diagnostic criteria do not always reflect experiences and may change. Diagnostic labels are significant in relation to illness identity^7^—so changing classification of experiences has personal and treatment impact. The merit of the diagnoses associated with SMI has been vigorously debated, with concerns that diagnostic labels and criteria inadequately reflect the experiences of those living with distressing feelings and beliefs.^8^, ^9^ Recovery is now on offer to—perhaps even an expected undertaking of^8^—all of those deemed to be seriously mentally ill.

The limited availability of non‐pharmacological approaches, and trained personnel to deliver them, continues to be an important barrier to appropriate care for many people with mental ill‐health.^10^ Mental health service users have described their experiences as being medicalized; understood through a medical framework and treated with medical interventions.^11^ Despite this, there are currently no methods available that reliably predict which treatment is going to work for which person, so selecting the right treatment is a challenge.^12^ The best care planning involves multidisciplinary staff, invites whole families to participate and considers whole lives.

Interventions which adopt person‐centred perspectives result in better clinical outcomes for people diagnosed with schizophrenia.^13^ Shared decision making (SDM) describes a model for health communication which encourages an egalitarian approach to decision making, recognizing the skills and experience offered by all participants involved in treatment decisions. There are ethical, clinical and economic arguments for SDM: only those in receipt of treatment can evaluate trade‐offs in relation to side‐effects, and service users experience better outcomes when provided with appropriate information and supported to make choices.^14^ Charles et al.^15^ outline key features of SDM

1. Collaborative working between at least the patient and provider
2. Sharing of information and exploration of health concerns
3. Discussion of treatment options and preferences
4. Agreed decisions about courses of action and implementation

Positive health outcomes associated with SDM include decreased hospitalization, improved satisfaction with treatment and adherence to medication.^16^ Service users diagnosed with SMI value opportunities to collaborate with care professionals and are prepared to engage with SDM within existing patient—professional relationships.^16^ However, much of the research conducted to evaluate the impact of SDM to date has focussed on patients with physical conditions, and little work has been conducted to explore SDM amongst people diagnosed with SMI or their families.

Adherence to prescribed medication has been a pre‐occupation for those researching the care of people with a SMI. A mean non‐adherence rate of 41% has been reported amongst those diagnosed with schizophrenia,^17^ and it has been suggested that 75% discontinue their prescribed medication within 18 months.^18^ The effects of non‐adherence to prescribed medication include reduced treatment efficacy,^19^, ^20^ increased risk of relapse^19^ and adverse health outcomes.^21^ Adherence to antipsychotic medication is an important predictor of illness course^22^ and could be a valid precursor to non‐medical approaches and longer‐term recovery. When considering the reasons for non‐adherence, many service users outline concerns about their medication, feel that prescribing decision making is not inclusive^23^ and have described feeling disempowered with doctors.^24^ Collaborative and trusting relationships between professionals and service users increase the possibility of SDM,^16^ enhance satisfaction and could improve adherence to care plans.^19^, ^25^ The complexity of decision making within mental health‐care commends a model of person‐centred care such as SDM to improve experience and concordance.

Despite the potential benefits of SDM, mental health professionals have been criticized for not involving service users or their family members in care planning.^26^ Research has found high rates of helplessness experienced by family members.^27^ Relatives have been found to have feelings of inferiority to staff which could explain silence from family members, often taken as acquiescence or acceptance by staff.^28^ Disparity is evident between professionals and service users in relation to the desired outcomes, with professionals placing greater emphasis on symptom reduction than service users.^6^, ^29^ Working alliance is vital to the success of SDM, reinforcing the active role required from professionals and service users.^16^ Much less is known about the preferences of family members, and there has been little research conducted to look at the views of family in relation to treatment preferences, their priorities and understandings of recovery, their support or educational needs. Eliacin et al.^16^ did not consider the views of family members; however, many of the quotes provided by service users prove illustrative of the central roles that family play in relation to recovery.

The involvement of family members within mental health care has been central to UK policy for 15 years, reflecting an international recognition of the importance of family support. Smith and Birchwood^30^ highlighted the “problem of engaging families in a therapeutic programme” as a major national issue. Partners in Care (RCPsych^31^) highlighted the problems faced by carers of people with different mental health problems, encouraging true partnership between carers, patients and professionals. The Carers Trust launched guidance relating to the “triangle of care” in 2010, updated in 2013. This approach acknowledges that models of engagement appear disconnected and recommends partnership working between service users, carers and organizations to achieve therapeutic alliance.^32^ In 2015, the National Involvement Partnership^33^ introduced national minimum standards for the involvement of carers in UK mental health services.

Barriers to involving family include unhelpful staff attitudes, unsupportive services, poor communication and inadequate information sharing.^34^ Families want to receive information that is tailored to their specific experience and needs, specifically explanations on how to carry out their caring role more effectively.^35^ Stigmatized attitudes towards individuals diagnosed with SMI have been found to adversely impact their mental health and well‐being. The “Time to Change” initiative was launched to address this stigma, and there have been significant improvements in public attitudes particularly relating to prejudice and exclusion.^36^ Despite this, nearly nine of 10 people with mental health difficulties say that stigma and discrimination have a negative impact on their lives.^37^ Courtesy stigma refers to the impact of stigma on people who are associated with those diagnosed with stigmatizing health conditions.^38^ Little is known about the impact of courtesy or direct stigma on the family members of people with SMI.^38^

Research and practice suggest there could be benefits derived from encouraging family members to adopt active coping strategies^5^ and that increased contact between health professionals and family members could decrease carer burden.^4^ However, there is little research to understand the extent to which family members wish to be involved in decision making, how, by whom and when the notion of involvement is introduced, and the roles adopted by (or assigned to) family in relation to decision making.

## 2 MATERIALS AND METHODS

This UK study was conducted within a large mental health and learning disability NHS organization.

Inclusion criteria:

1 Staff
  - Current member of staff within the recruiting organization
  - Registered prescriber (medical or non‐medical)
  - Working within adult mental health services
2 Family
  - Providing informal care for a service user currently in receipt of adult mental health services within the recruiting organization.
  - Capacity to give informed consent

Non‐prescribing staff were excluded as the study was designed to explore the views of those with prescribing rights towards the role of family within SDM, to consider family input to decisions about medicines (as a first‐line treatment). Staff working within specialist dementia or memory clinics were excluded, as were staff working within child services.

Previous work within this region has experienced difficulties with face‐to‐face methods of data collection due to travel constraints. To overcome this, qualitative feedback was gathered on a written questionnaire, distributed in hard and electronic copies. Questions were designed to give participants the opportunity to write detailed responses, including unprompted entries. Question topics were identified from a literature search. Additional questions were informed from anecdotal feedback and input from a project steering group (funder, research team, carer representative, Research & Innovation (R&I) lead). A draft survey was circulated to the steering group then piloted with two colleagues. Topics included the following:

1 Experiences of involvement to date
  - Attendance at appointments
  - How involvement was instigated/encouraged / prevented
  - Information exchange—resources about treatment / diagnosis / potential involvement
2 Participation in decision making during appointments or care more generally
  - Perceived role of involvement
  - Resolving conflict or different opinions
3 Facilitators and barriers to involvement

The areas of questioning did not change post‐piloting, but the wording of specific questions was amended to enhance readability.

The study was granted ethical permission from the University ethics committee and given formal approval by the relevant R&I department.

## 3 RESULTS

Carer participants were recruited through snowball and opportunity sampling. A member of the research team (DG) attended four carer meetings and one carer workshop to introduce the study, taking hard copies of the survey tool with a sealable box for return, and the link to the e‐survey tool. Posters to promote the study were placed across the organization. Information about the study and the e‐survey link was sent to 16 carer groups within the region. This information was also sent to 19 members of staff who identified as leads for carer involvement. Given the wide distribution of invitations to family members, including posters, it is not possible to estimate how many family members considered participating in the study or to provide a response or refusal rate.

Forty‐six family member participants completed the survey questions, 30 females (65%) and 16 males (35%). Of these, 31 (67%) were completed on a paper version then entered onto the e‐version by the research team. The high proportion of responses completed in hard copy amongst this group reflects in part the benefit of having a research project introduced to participants in person, to provide support with completion or answer questions about the study.

Carer respondents were all family members. The age of participants ranged from 18 to 80 years, with the majority of participants aged between 61 and 65 years (n=9; 20%). Seventeen were caring for a child over 18 years and two for a child under 18 years. Nineteen were caring for a partner/spouse (husband n=7, wife n=6, partner n=4, fiancé n=1 and other n=1). Four participants were caring for a sibling, and two were involved in the care of a parent. Two declined to specify their relationship.

The diagnoses of family members were not always known or disclosed but those outlined included schizophrenia (n=3), bipolar disorder (n=1), autistic spectrum (n=1) and Aspergers (n=1). In addition to these, one of the participants who did not disclose a diagnosis subsequently wrote about their experiences of caring for a daughter with personality disorder. The high number of instances where diagnoses were unknown or not disclosed could suggest a lack of family involvement or knowledge, the use of working diagnoses in practice or reluctance to utilize/share diagnostic labels.

A total of 158 members of staff were identified as eligible for participation and emailed information about the study including a link to the e‐survey. A reminder email was sent 2 weeks later. Paper copies of the survey were left in ward offices and with medical secretaries, with sealed envelopes for return. Surveys were taken by the research team to a non‐medical prescriber meeting, with sealable envelopes. Fifty‐five members of staff completed the survey (response rate=35%), including 33 doctors (60%) and 22 nurse prescribers (40%). Of these, 19 responses (35%) were completed in hard copy then entered onto the online tool by the research team.

A thematic analysis of the qualitative feedback was undertaken by the two authors. Written comments were analysed using Excel to “hide” the group membership of participants. Once theming was complete, it was possible to reveal group membership and compare themes within and across participant groups. Techniques of thematic analysis were used,^39^ including the early identification of concepts from written comments for comparison and contrast across instances.^40^ Concepts were grouped together as themes with member checking across both the research team and the project steering group. At the end of the analysis, overarching themes included the following: Defining Involvement and Rules of Engagement.

### 3.1 Defining involvement

Both participant groups defined family involvement as a process of “opinion‐seeking”—to shape decisions about treatment options but also to consolidate decisions proffered by staff

By involved *I mean I was asked my opinion* on how I thought he was progressing

(Family, 18685)

Not changed a decision but consolidated a decision through their encouragement and approval

(Staff, 18833)

For family, involvement represented an opportunity to be “listened to,” an opportunity which was fully realized when their views were heard, then incorporated

To be listened to and my opinions valued and my safety considered

(Family, 18570)

Chance to talk about my concerns and what was happening

(Family, 19026)

Such comments reiterate those highlighted by Rowe.^34^ When family members adopt active coping strategies and make a practical contribution, they reduce their levels of distress.^5^ The supportive function of involvement was recognized in this study

They have supported me and involved me in every aspect of my partner's treatment. At no point have I felt that they have considered my feedback or feelings irrelevant

(Family, 19050)

### 3.2 Rules of engagement

**Figure 1:**
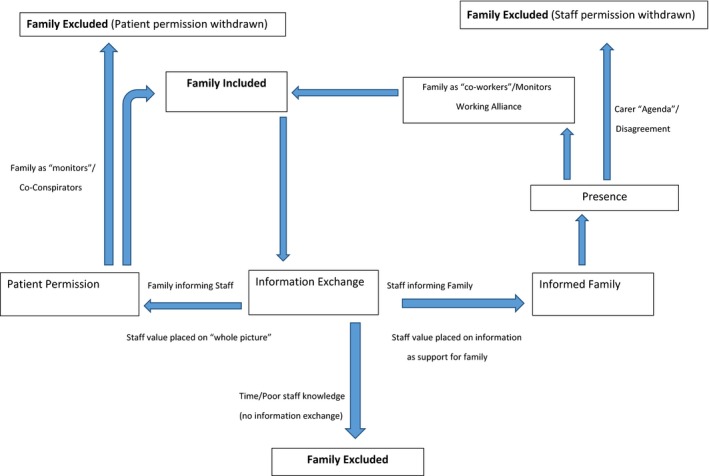
Rules of engagement

Interviews highlighted a number of implicit rules at play prior to and during family involvement, which resulted in family being either included in, or excluded from, opportunities for shared decision making. The overarching theme of “Rules of Engagement” makes explicit these rules and contains a number of subthemes; Patient Permission, Presence, Information Exchange, Monitoring and Staff Permission / the Carer “Agenda.” The subthemes are explicated below, and relationships between them are highlighted in a conceptual memo (see Figure 1).

#### 3.2.1 Patient permission

Participants were aware that patient permission had to firstly be navigated, and it was felt, by both groups, that patient views were prioritized

Patient is given the choice always to get their relatives involved at any stage. I do not push at the initial stages as I want to build a trusting relationship first

(Staff 18802)

Communication with professionals involved, without confidentiality getting in the way. It can be got around without conveying confidentiality. A common sense approach is needed

(Family 19043)

There was a lack of certainty about Patient Confidentiality Policies and, as with previous research,^31^ it was felt that staff anxiety about contravening confidentiality could exclude family from staff contact, prevent information exchange and, ultimately, deny family the opportunity for involvement. Patient permission for family contact is not only navigated by staff at early appointments, but revisited by staff at multiple care points. Permission may be withdrawn at any time, if a family—service user relationship deteriorates, or due to service user concerns about information exchange between family and staff.

#### 3.2.2 Presence

For staff, the next “rule” was fulfilled by the physical presence of family members at appointments:

If a carer or other family member is present they will generally be involved in the discussion about medication

(Staff 18529)

If a carer is present at a care plan review then I would ask their opinion, however most of my service users attend reviews independently

(Staff 19394)

If they are present I always do it, if not, then I can't

(Staff 19060)

This suggests staff adopt a passive approach to family engagement, waiting for family to attend appointments, rather than actively encouraging attendance by sharing information or negotiating meeting times. Without active attempts to inform family, staff are playing into notions of silence as acquiescence or acceptance.^28^

Family participants reflected this by highlighting the challenges of “getting‐in” to appointments and being “given a chance” by staff. Two of the family participants described how they had to take opportunities themselves:

I made myself involved in the care and decisions made about my wife I wasn't asked

(Family 19027)

By taking steps to involve myself of the working on our Trust, of mental health legislation, of research papers on enduring mental health, of the wider world of mental health, in short—knowledge

(Family 18302)

Only families holding relevant information about service policy and practice would be able to “get in” to appointments in this way, reinforcing the important role of staff in sharing such information. In addition, decision making commonly takes place at review meetings which may be 1‐1 or conducted with multidisciplinary teams (MDTs). To participate in these meetings, family members needed to be present, but their presence was determined or moderated by staff invitation.

#### 3.2.3 Information exchange

Both participant groups recognized that information was an essential precursor to family involvement; however, staff time constraints reduced opportunities for information exchange between family and staff, excluding family from possible involvement.

Staff participants emphasized the important role played by family in providing wider, contextual information about the service user, to enhance understanding and inform decision making:

[Family] often help to give a wider view of patients’ personality, attitude to treatment, response to treatment, compliance

(Staff, 18568)

Gain understanding on family norms and beliefs about treatment and mental health

(Staff, 18718)

In addition, staff participants felt that family had a key role in terms of providing context‐specific information about service user response to, and adherence with, prescribed medication. A possible downside of this was that some staff members conceptualized family involvement through a predominantly practical lens—what carers can “do”—rather than a holistic consideration as related to either hopefulness^6^ or recovery.

For family, information exchange served a dual purpose—to shape decisions, but also to support them, as carers.^35^ The opportunity to provide information to staff, to have a platform in which to share thoughts and views about care, and to be listened to, was a key supportive resource for family participants:‐

To be listened to and my opinions valued and my safety considered

(family 18570)

To be listened to by people who did not know her. Not to be treated as irrelevant

(family 18680)

Information was sought by family as a means by which to enhance their mental health literacy and capacity to support at home:

More information about illness. More help with problems faced

(Family, 19052)

More information on how mental health treatments work

(Family, 18569)

Staff recognized the importance of information for family, describing a number of strategies undertaken to share information when family members were not present at appointments:

Often partners are not present however I give information about the medication for women to take home and discuss with their partners if they wish to

(Staff, 18567)

Many service users attend by themselves so I cannot actively involve carers in that setting however I provide patients with handy charts to take away to discuss with carers if they choose

(Staff, 18767)

Relationships with staff played a key role in terms of enabling family to share information with whole health‐care teams. In the presence of good relationships, information exchange progressed and some family members became recognized by staff as core team members/co‐worker:

Helpful for them to understand the medication—monitor and support its use, and offer feedback on changes in their loved one for better / worse around treatment response—as a sort of co‐therapist can help around diet, smoking, exercise as well as emotional and practical support

(Staff, 18811)

Building a relationship with members of the care team. Regular contact with a key‐nurse during inpatient stays…

(Family, 18344)

#### 3.2.4 Monitoring

Some staff were keen to involve family in decision making as they recognized that family had influence and could adopt reassuring or potentially persuasive roles:‐

Carers often have good influence on motivating clients to change or look at things differently and the client is more likely to take these changes on board and maintain them with professional and carer involvement /support. They may consider alternative treatment options more readily

(Staff, 18534)

I have had a partner encourage his wife to take medication as she was very distressed and didn't want to make the decision alone

(Staff, 19060)

There was a particular emphasis placed by staff, but not family participants, on the role of family as “medication monitors,” to overview compliance, assess treatment impact and assert influence

When family are heavily involved in their care, maybe they help to remind the person to take their medication

(staff, 18535)

They can help with compliance, if support is there they are more likely to take the medication. If the medication is sedating the partner needs to support more

(Staff, 18567)

Staff emphasized the important role for carers in ensuring that service users follow treatment advice (commonly the taking of medication) and strive for a recovery as defined by their health team (the reduction of symptoms).

This focus on adherence, particularly medication adherence, could be problematic for family members who felt that discussions became medicalized:

The discussions are about how my son is generally functioning, if he is regularly taking his prescribed medication and most importantly if the support services are monitoring his medication regime (taking and reordering of medication etc)

(Family, 18532)

For family members, adopting or being assigned a “medication monitor” role could also be problematic. Family reflected on instances whereby sharing their opinion about progress and /or adherence was perceived by service users as an alliance between staff and family, rather than a whole team alliance:

…. I was asked my opinion on how I though he was progressing. This was difficult as I either said the truth that he was very ill and paranoid, but this risked alienating my son who would then have thought I was part of the conspiracy

(Family, 18685)

When family members are encouraged to report on progress, there is the possibility that their identities at home change to coconspirators, not co‐workers. The inherent risk was that family loyalties would be tested if opinions about perceived progress varied and service users may then withdraw permission for their involvement‐

My son won't let me attend anymore because I agree with the Consultant Psychiatrist that he SHOULD remain on medication

(Family, 19388)

#### 3.2.5 Staff “permission” and the carer “agenda”

There was a sense from some family participants that staff perceived them in a negative light and actively excluded them

We have been excluded from review meetings in the past and not given necessary information about decisions made about our son. I was accused by a previous care co‐ordinator of causing mental health problems for my son because of my own anxieties

(Family 18532)

I was given the impression that my input was not welcomed and possibly resented as interference which I fail to understand as being a carer I need to know and understand what the overall picture and future is the aims

(Family 18371)

The findings also suggest that some staff felt challenged by family members who held different, and possibly antagonistic, views to staff and this could lead to them being excluded from decision making

Suspicion that carer may not have best interest of SU

(Staff 18819)

If I felt that the carer was not acting in the best interests of the service user. But this would be the exception rather than the norm

(Staff 18695)

Carer not making ‘best interest’ decisions

(Staff 19394)

It was not possible to identify from this study how such conclusions were drawn, but this is a barrier to shared decision making and family involvement which warrants further exploration in terms of the attitudes of staff towards family.

## 4 DISCUSSION

This study suggests that staff value the contextual information that family can provide, particularly at points of decision making. Despite this, family felt the information they shared with teams remained on the periphery of decision making. Rather than being a central tenet of care decisions made, information was primarily sought from family in order to highlight opinions about care or to consolidate those decisions already made by the care team, particularly in terms of delivering care at home. Taking into account the Hickey and Kipping^41^ model of user involvement, this reflects a view of family as consumers rather than democratic members of the care team. In terms of SDM more specifically, opportunities became available to family through staff who acted as gatekeepers to involvement, moderating the potential for family to act collaboratively, rather than opening up potential involvement in decision making for all family in contact with services.

Staff have an increasing awareness of their responsibility to inform, if not fully involve, family in care planning and treatment decisions. Given the increasing focus placed on the role of SDM within health care generally, this could suggest a shift within adult mental health care from explicitly paternalist models of decision making, towards informed decision making. There is no suggestion from this study that SDM has yet been fully integrated within routine mental health‐care practice.

Staff in this study predominantly highlighted the role of information from family in terms of monitoring adherence and service user “progress.” In terms of staff recognizing the important role of family in this respect, this acted as a facilitator to family involvement. However, for family members, this was not a neutral role and could result in conflict with service users and, sometimes, subsequent exclusion (via patient refusal). This suggests a need for additional support and training for those involving family members in reviews and decision making to raise awareness about these risks.

Good relationships between family members and other health‐care team members were important facilitators to family input—as with SDM more broadly—respectful, working alliances^13^ facilitated family involvement. Team communication is important, to encourage staff to fully evaluate instances where service user permission is not granted, or to discourage staff from discounting family input due to caution about a possible carer “agenda.” Staff have a responsibility to prevent family members being subject to “courtesy stigma”^42^ or direct stigma from those who may hold the view that pathology is solely rooted in family relationships and dynamics.^43^ Named key workers or peer advocates/recovery workers could facilitate family involvement by actively negotiating co‐worker roles and ascribing agreed responsibilities.

Making explicit the “rules of engagement” for family input, heightening awareness of the barriers, increasing awareness of policy (including patient confidentiality policies) and disseminating the potential benefits of family input would be important first steps in terms of encouraging staff to further consider family involvement as a core constituent of shared decision making.

### 4.1 LIMITATIONS

Due to the recruitment strategy for this study, it would be inappropriate to infer that these findings are representative of family experience broadly. Some family participants shared experiences where they had perceived active exclusion from staff just as some acknowledged the important role that staff played in supporting them.

Only staff members with prescribing authority were invited to participate and of these only 35% did participate. This is a relatively low response rate, and the findings should be interpreted with this in mind. Future research will include mental health nurses and care coordinators to encourage a broader discussion of family involvement. The self‐selected nature of recruitment could mean that participants holding strong opinions about family involvement (negative or positive) were overrepresented. Despite this, the range of views collected and clustering of themes would suggest that findings are trustworthy. It was not possible to include non‐English speakers within the study, but this is acknowledged as an important area for future study.

## 5 CONCLUSIONS

This study suggests that staff have an increasing awareness of the need to inform family and to move towards a model of informed, if not yet shared, decision making. Family has unmet needs in relation to information, which can serve as a supportive and practice resource. Adherence to medication continues to be a pre‐occupation for prescribing staff, who respond by assigning “monitoring” roles to carers. The prioritization of adherence should be challenged and staff could be encouraged to consider the broader nature of medicines optimization, including the important roles played by whole families when optimizing treatments. Such challenge could also support staff to consider broader roles for family, to negotiate beyond family roles which exclusively focus on and reward, “medication monitoring.” In accepting such a challenge, whole teams should consider the difficult position in which family members find themselves, in relation to their caring roles and responsibilities.^44^

There are a number of steps prior to family involvement, and subsequent SDM, which include the seeking of service user permission, and timely sharing of information. Both these steps are regulated by staff so it is important to share information with clinical teams about the possibilities of family involvement and to deter service‐centred, rather than person‐centred, delivery. Staff would benefit from additional training in relation to patient confidentiality, particularly as related to information exchange with family.
